# Chronic vagus nerve stimulation for drug-resistant epilepsy may influence fasting blood glucose concentration

**DOI:** 10.1186/s12938-020-00784-1

**Published:** 2020-05-29

**Authors:** Hongyun Liu, Ping Zhan, Fangang Meng, Weidong Wang

**Affiliations:** 1 Research Center for Biomedical Engineering, Medical Innovation & Research Division, Chinese PLA General Hospital, Beijing, 100853 China; 2 Center of Medical Device R & D and Clinical Evaluation, Chinese PLA General Hospital, Beijing, 100853 China; 3 Beijing Neurosurgical Institute, Beijing, 100050 China; 4 Neurosurgery, Beijing Tian Tan Hospital Capital Medical University, Beijing, 100050 China

**Keywords:** Blood glucose, Glycemic control, Vagus nerve stimulation, Autonomic nervous system, Glucose homeostasis

## Abstract

**Background:**

Cervical vagus nerve stimulation (VNS) has been widely accepted as adjunctive therapy for drug-resistant epilepsy and major depression. Its effects on glycemic control in humans were however poorly understood. The aim of our study was to investigate the potential effects of VNS on fasting blood glucose (FBG) in patients with drug-resistant epilepsy.

**Methods:**

Patients with drug-resistant epilepsy who had received VNS implants at the same hospital were retrospectively studied. Effects on FBG, weight, body mass index and blood pressure were evaluated at 4, 8 and 12 months of follow-up.

**Results:**

32 subjects (11 females/21 males, 19 ± 9 years, body mass index 22.2 ± 4.0 kg/m^2^) completed 12-month follow-up. At the 4 months, there were no significant changes in FBG concentrations from baseline to follow-up in both Sham-VNS (4.89 ± 0.54 vs. 4.56 ± 0.54 mmol/L, *N* = 13, *p* = 0.101) and VNS (4.80 ± 0.54 vs. 4.50 ± 0.56 mmol/L, *N* = 19, *p* = 0.117) groups. However, after 8 (4.90 ± 0.42 mmol/L, *N* = 32, *p* = 0.001) and 12 (4.86 ± 0.40 mmol/L, *N* = 32, *p* = 0.002) months of VNS, FBG levels significantly increased compared to baseline values (4.52 ± 0.54 mmol/L, *N* = 32). Changes in FBG concentrations at both 8 (*R*^2^ = 0.502, *N* = 32, *p* < 0.001) and 12 (*R*^2^ = 0.572, *N* = 32, *p* < 0.001) months were negatively correlated with baseline FBG levels.

**Conclusions:**

Our study suggests that chronic cervical VNS elevates FBG levels with commonly used stimulation parameters in patients with epilepsy.

*Trial registration* VNSRE, NCT02378792. Registered 4 March 2015—Retrospectively registered, https://clinicaltrials.gov/ct2/show/NCT02378792

## Background

Cervical vagus nerve stimulation (VNS) is widely accepted as an adjunctive therapy for drug-resistant epilepsy and major depression that involves intermittent electrical stimulation of the left vagus nerve by a subcutaneously implanted programmable pulse generator. Since the first human implant of VNS therapy in 1988, more than 100,000 patients have been treated with VNS worldwide by the end of 2019 [1]. The experimental data about VNS in treatment of sepsis and stroke, clinical trial data about VNS in treatment of heart failure, chronic pain, obesity, and tinnitus indicate that VNS therapy may have many potential indications [2–8]. To date, however, the exact changes in blood glucose concentrations in patients undergoing VNS treatment remain largely unknown.

VNS was reported to have mixed influences on blood glucose levels in previous studies based on animal models. Early work in anesthetized dogs indicated that stimulation of the ventral vagus nerve resulted in no statistically significant change in plasma glucose level [9]. Data from the insulin sensitivity study suggested that left cervical VNS yields an increase in blood glucose concentration with an increase in plasma insulin immunoreactivity in fed anesthetized rats [10]. Meyers et al. also demonstrated that electrical stimulation of the intact or afferent cervical vagus nerve in rats causes a marked and sustained increase in blood glucose levels by suppression of pancreatic insulin secretion [11]. In addition to confirming that chronic VNS raises blood glucose concentrations, a recent animal trial also revealed that cervical VNS impairs glucose tolerance [12]. In contrast, data from a previous study showed that efferent cervical VNS lowered blood glucose concentrations by activation of insulin secretion [11], whereas analysis of the study conducted in obese pigs indicated that chronic bilateral vagal stimulation has the capacity to restore fasting glucose metabolism [13]. Similarly, Yin et al. found VNS at low frequency resulted in a hypoglycemic effect in both normal and diabetic rats by enhancing vagal efferent activity [14]. However, although the studies in animal models suggest that acute or chronic VNS has potential effects on glycemic control and glucose metabolism, far too little attention has been paid to the possible influences of chronic cervical VNS on fasting blood glucose (FBG) levels in humans.

To the best of our knowledge, nothing has been reported in the literature on cervical VNS on human FBG concentrations. The aim of the present study was to broaden the current insight on cervical VNS-induced influence on human blood glucose control. We retrospectively analyzed data from one center of a randomized, double-blind controlled trial to assess the efficacy and safety of 12 months of VNS in patients with drug-resistant epilepsy. The primary objective of this study was to assess the effects of chronic VNS on FBG levels across different follow-up time points. Moreover, we examined the association between FBG level and its changes at follow-up period and selected clinical variables such as body mass index (BMI), blood pressure and stimulation parameters.

## Results

### Participants and demographics

The baseline demographic data, clinical characteristics, and preoperative diagnosis are given in Additional file 1: Table S1. The 32 patients included 21 men and 11 women ranging in age from 6 to 38 years at the time of VNS implantation. There were 28 patients whose seizures responded to VNS, with 17 patients having seizure reductions of at least 50% at the end of the 12-month follow-up period. After the double-blind period, 13 patients in the sham-VNS group also started receiving VNS treatment. A total of 5 (3 of them were from the VNS group, and the other 2 were from the sham-VNS group) of the 32 patients became seizure-free after the 12-month VNS treatment with no serious adverse events reported in the study population.

### Effects of VNS on FBG concentration

**Fig. 1:**
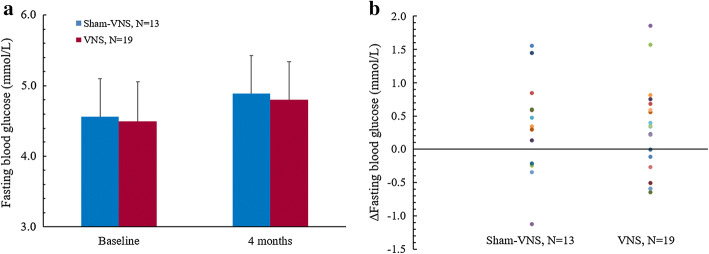
FBG concentration and its changes in sham-VNS and VNS groups. **a** FBG levels at baseline and at 4-month follow-up in the two groups. **b** Changes in FBG levels at 4-month follow-up in Sham-VNS and VNS groups. There were no significant differences in FBG levels between Sham-VNS and VNS groups. The FBG levels at 4-month follow-up were not significantly different from baseline values in both groups

Demographic data and clinical factors of the VNS group and the sham-VNS control group are presented in Additional file 1: Table S2. At baseline, there were no significant differences in gender distribution, age, AED regimens and seizure characteristics between the two groups. At the 4-month double-blinded follow-up period, FBG levels significantly increased in the overall study population (*N* = 32) compared to baseline (4.83 ± 0.53 mmol/L vs. 4.52 ± 0.54 mmol/L, *p* = 0.026). However, this overall change in FBG concentration was weakened when considering the VNS (*N* = 19) and sham-VNS (*N* = 13) groups separately. As shown in Fig. 1, a comparison of the VNS and sham-VNS groups indicated no significant difference in FBG concentration (4.50 ± 0.56 mmol/L vs. 4.56 ± 0.54 mmol/L, *p* = 0.969) between groups over the course of the double-blinded period. In addition, the FBG levels at 4-month follow-up were not significantly different from baseline values in both groups (VNS: 4.80 ± 0.54 mmol/L vs. 4.50 ± 0.56 mmol/L, *p* = 0.101; sham-VNS: 4.89 ± 0.54 mmol/L vs. 4.56 ± 0.54 mmol/L, *p* = 0.117).

**Fig. 2:**
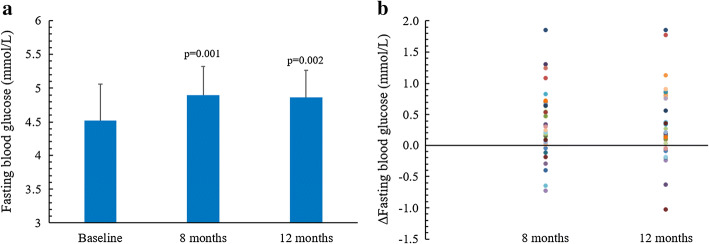
FBG concentration and its changes for the 32 patients receiving VNS treatment. **a** FBG levels at baseline, 8, and 12 months of long-term follow-up. **b** Changes in FBG levels at 8- and 12-month follow-up in the study subjects (*N* = 32). Significant differences were noted in FBG concentrations for patients receiving VNS treatment at both 8 months’ (*p* = 0.001) and 12 months’ (*p* = 0.002) follow-up compared to baseline values

There were no significant differences in FBG levels between the VNS and sham-VNS control groups at 8-month (4.87 ± 0.48 mmol/L vs. 4.94 ± 0.33 mmol/L, *p* = 0.715) and 12-month (4.90 ± 0.42 mmol/L vs. 4.79 ± 0.38 mmol/L, *p* = 0.477) follow-up. As shown in Fig. 2, FBG levels significantly increased in the 32 subjects for both 8-month (4.90 ± 0.42 mmol/L vs. 4.52 ± 0.54 mmol/L, *p* = 0.001) and 12-month (4.86 ± 0.40 mmol/L vs. 4.52 ± 0.54 mmol/L, *p* = 0.002) visits compared to baseline values. In specific, both the VNS (4.87 ± 0.48 mmol/L vs. 4.50 ± 0.56 mmol/L, *p* = 0.018) and sham-VNS (4.94 ± 0.33 mmol/L vs. 4.56 ± 0.54 mmol/L, *p* = 0.021) groups had a significant increase in FBG concentrations at 8 months as compared to baseline. At 12 months, patients in the VNS group had a significant increase in FBG level as compared to baseline (4.90 ± 0.42 mmol/L vs. 4.50 ± 0.56 mmol/L, *p* = 0.002), whereas patients in the sham-VNS group did not (4.79 ± 0.38 mmol/L vs. 4.56 ± 0.54 mmol/L, *p* = 0.221).

### Effects of VNS on body weight, bmi, and blood pressure

There were no significant differences in body weight, BMI, systolic blood pressure and diastolic blood pressure between the VNS and sham-VNS groups. Body weight, BMI and blood pressure at various clinical visits following VNS device implantation are shown in Tables 1 and 2. At 4 months, there were no significant changes in body weight, BMI, systolic and diastolic blood pressure compared to baseline. No significant differences were observed for changes in systolic and diastolic blood pressure at 8- and 12-month follow-up. For the overall study subjects, there was an increase in BMI at 12-month (23.1 ± 4.3 kg/m^2^ vs. 22.2 ± 4.0 kg/m^2^, *p* = 0.009) follow-up compared to baseline. Moreover, measures of body weight increased both over the course of 8 (59.9 ± 20.4 kg vs. 57.8 ± 20.4 kg, *p* = 0.001) and 12 (61.7 ± 19.9 kg vs. 57.8 ± 20.4 kg, *p* = 0.006) months in the study population compared to the preoperative weight. Weight gains at 8 and 12 months were 2.1 ± 0.7 kg and 3.9 ± 0.7 kg, respectively.

**Table 1:** Baseline and 4-month follow-up characteristics of the Sham-VNS and VNS groups

Variables	Sham-VNS, *n* = 13		VNS, *n* = 19		p1	p2	p3
	Baseline	4 months	Baseline	4 months			
Height (cm)	162 ± 16	163 ± 15	155 ± 23	157 ± 21	0.700	0.041	0.009
Body weight (kg)	60.9 ± 20.4	61.1 ± 19.2	55.6 ± 20.8	57.1 ± 21.0	0.282	0.685	0.065
BMI (kg/m^2^)	22.3 ± 3.9	22.2 ± 3.7	22.2 ± 4.2	22.3 ± 4.3	1.000	0.814	0.199
Systolic blood pressure (mmHg)	116 ± 21	114 ± 13	115 ± 14	115 ± 12	0.788	0.666	0.896
Diastolic blood pressure (mmHg)	73 ± 11	74 ± 8	76 ± 11	78 ± 10	0.847	0.944	0.081

*p* values were provided for comparison as follows: (1) p1: Sham-VNS vs. VNS at baseline; (2) p2: Baseline vs. 4-month follow-up for Sham-VNS group; (3) p3: Baseline vs. 4-month follow-up for VNS group

**Table 2:** Characteristics and VNS settings of the 32 patients with epilepsy at baseline, 8 months’ and 12 months’ follow-up

Variables	Baseline	8 months	12 months	p1	p2	p3
Height (cm)	158 ± 20	160 ± 18	159 ± 18	0.003	0.011	0.432
Body weight (kg)	57.8 ± 20.4	59.9 ± 20.4	61.7 ± 19.9	0.001	0.006	0.006
BMI (kg/m^2^)	22.2 ± 4.0	22.5 ± 4.2	23.1 ± 4.3	0.294	0.009	0.054
Systolic blood pressure (mmHg)	115 ± 17	111 ± 11	113 ± 12	0.094	0.318	0.288
Diastolic blood pressure (mmHg)	75 ± 11	73 ± 11	75 ± 9	0.436	0.658	0.260
Amplitude (mA)	–	1.3 ± 0.5	1.5 ± 0.4	–	–	< 0.001
Pulse width (μs)	–	500 ± 0	500 ± 0	–	–	1.000
Frequency (Hz)	–	30 ± 0	30 ± 0	–	–	1.000
On time (s)	–	30 ± 0	30 ± 0	–	–	1.000
Off time (min)	–	5 ± 0	5 ± 0	–	–	1.000

*p* values were provided for comparison as follows: (1) p1: after 8-month VNS vs. baseline; (2) p2: after 12-month VNS vs. baseline; (3) p3: after 12-month VNS vs. after 8-month VNS for all the study subjects

### Effects of age, BMI, baseline FBG levels, and stimulation amplitude

**Fig. 3:**
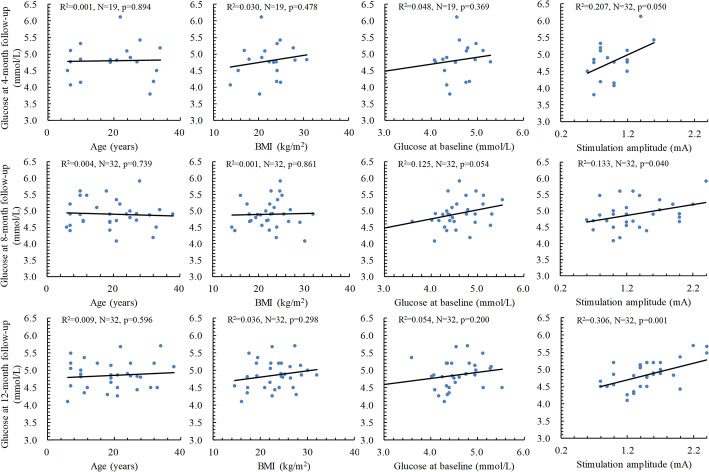
Correlations between FBG levels at follow-up and BMI at follow-up, age, and FBG levels at baseline. The straight lines and the statistics (*R*^2^, *N, p*) are for the linear correlations and regressions between the two parameters with the study subjects according to the multiple linear regression analysis

**Fig. 4:**
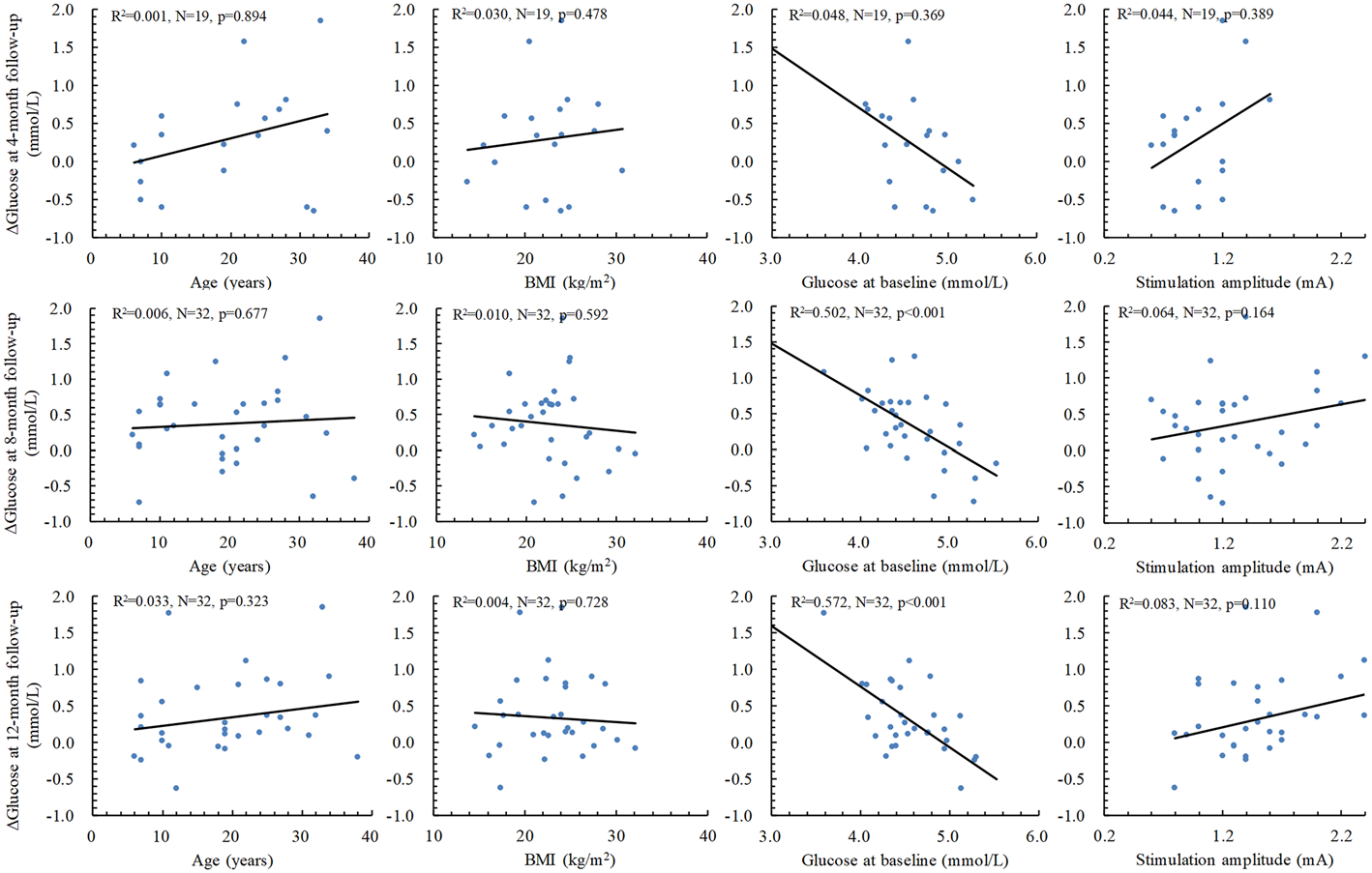
Effects of age and FBG levels at baseline, BMI, and stimulation amplitude at corresponding follow-up period on changes in FBG levels from baseline to different follow-up periods. The straight lines and the statistics (*R*^2^, *N*, *p*) are for the linear correlations between the two parameters with the study subjects according to the multiple linear regression analysis

Multiple linear regression analysis showed that age at VNS implantation, baseline fasting glucose concentrations, and BMI at corresponding visits were not identified as significant predictors for FBG levels at various follow-up time points (Fig. 3). For the overall study population, higher stimulation amplitude was associated with higher FBG levels at 8 (*p* = 0.040) and 12 (*p* = 0.001) months’ follow-up. Partial correlation also demonstrated that there were moderate, positive partial correlation between FBG levels and stimulation amplitude while controlling for BMI (31.1 ± 9.1 years) at 8 (*r* = 0.364, *N* = 32, *p* = 0.044) and 12 (*r* = 0.544, *N* = 32, *p* = 0.002) months of follow-up. As shown in Fig. 4, multiple linear regression results indicated that changes in FBG values (*p* < 0.001). In addition, age at baseline, BMI, and stimulation amplitude at the corresponding follow-up period had no significant effects on changes in FBG concentrations from baseline to various follow-up visits.

## Discussion

The present retrospective study demonstrated for the first time that chronic cervical VNS may have the potential ability to increase FBG in patients with drug-resistant epilepsy. However, the elevated FBG levels which evolved the course of 12 months are still within the normal range. The changes in FBG concentrations did not associate with age at baseline, BMI, and stimulation amplitude at the corresponding follow-up period. In addition, results showed that 12-month chronic cervical VNS significantly increased body weight of patients with drug-resistant epilepsy without obviously affecting their systolic and diastolic blood pressure.

The autonomic nervous system (ANS), which composed of sympathetic and parasympathetic (vagal) branches, is crucial in regulating glucose metabolism [15–18]. Furthermore, the central nervous system (CNS) maintains glucose homeostasis through ANS-mediated control of the metabolically relevant organs including pancreas, liver, gut, skeletal muscle, and brown and white adipose tissues [19, 20]. As a fast-growing neuromodulation technology in the past 30 years, cervical VNS has been proven to be a useful adjunctive therapy across a number of diseases by stimulating the mixed autonomic vagus nerve in the neck. Since the vagus nerve provides an extensive efferent and afferent network of innervation for the organs and glands related to glucose metabolism, whether cervical VNS will affect the patient’s blood glucose level in the treatment of drug-refractory epilepsy is a question worth studying. The most important observation of the present study shows that electrical stimulation of the left cervical vagus nerve, which provides a way to affect the autonomic function and regulate the autonomic tone, induces an elevation in FBG levels in patients with drug-resistant epilepsy. In fact, chronic VNS yields an increase in FBG concentrations in most patients, and consequently, the overall effect of VNS could be noticed. This finding is inconsistent with the results of a previous similar retrospective study, in which the conclusion is that chronic cervical VNS in patients with epilepsy is unlikely to increase blood glucose levels or induce glucose intolerance with frequently used stimulation parameters [21]. Nonetheless, results of the previous study varied widely, and further analysis suggests that stimulation on times longer than 25 s appears to be related to elevation in blood glucose levels, especially if the stimulation off time is shorter than 200 s. In our study, the stimulation on times for all patients was 30 s, and the corresponding results seem to confirm the previous concern that chronic cervical VNS increases blood glucose concentrations to a certain extent. Recently, different VNS methods including different stimulation modes and stimulation locations have been developed and applied to the study of glycemic control in humans. Stimulation mode varied from invasive to non-invasive; stimulation locations include cervical vagus nerve, subdiaphragmatic vagus nerve, and auricular vagus nerve. A pilot study in subjects with impaired glucose tolerance demonstrated transcutaneous auricular VNS (taVNS) significantly reduced 2-h glucose tolerance, which suggests that taVNS may be used as a preventive treatment for pre-diabetes [22]. Stimulating different locations by different stimulation modes (taVNS vs. cervical VNS) may cause activation of different nerve fibers, and therefore have disparate potential effects on glucose metabolism. In a series of studies focusing on electrical stimulation of anterior and posterior intra-abdominal vagal trunks for obese subjects with type 2 diabetes mellitus, vagus nerve blocking significantly decreased glycosylated hemoglobin (HbA1C), and the beneficial effect is sustained at 2-year follow-up period [23–25]. In addition, fasting plasma glucose concentrations were also significantly declined due to the effects of VNS. However, in a prospective pilot vagus nerve blockade study on 7 non-diabetic obese subjects, electrical stimulation of intra-abdominal vagal trunks showed no obvious beneficial effect on glucose metabolism, insulin secretion and action, or gastric emptying [26]. Combining the results of the above studies, we speculate that whether VNS affects blood glucose metabolism may have a potential relationship with the subject’s baseline blood glucose levels. In addition to the differences in stimulation mode, stimulation locations, and specific stimulation parameters, the research subjects of the present study are different from those previous related studies. The non-diabetic epileptic patients in our study experienced an elevation in FBG levels after 12-month VNS treatment. This may not be true for patients with diabetes mellitus since VNS may induce changes in appetite, energy regulation as well hormones such as incretins and GLP-1 that are associated with insulin secretion and glucose regulation [23, 24]. Furthermore, long-term increase in FBG concentrations is negatively associated with baseline FBG concentrations, which confirms our speculation and suggests that the effects of VNS on glycemic control are not a simple binary response process.

Our clinical observations remain inconsistent with experimental work demonstrating the effects of VNS on glycemic control in animal models. Continuous vagal stimulation was accompanied by a rise in blood glucose concentration and was reported in anesthetized dogs [27, 28]. The data from another early observational study showed that VNS in the dogs produces a moderate increase of glucagon secretion and subsequently increases the arterial glucose levels [29]. It is inferred that the effects of VNS on both the glucagon and insulin are mediated by ganglionic, nicotinic transmission. In a prandial insulin sensitivity study, VNS on fed rats caused an immediate increase in blood glucose levels, whereas a decrease in blood glucose level was observed after atropine administration [10]. However, cervical VNS had no significant effects on blood glucose levels and plasma insulin in fasted rats. In Zucker diabetic fatty rats, taVNS reduced the glucose concentration to a normal level in 7 days and effectively maintained the normal glycemic and plasma HbAlc levels when applied for 5 consecutive weeks [30]. Importantly, the antidiabetic effect of taVNS is related to the triggering of tidal secretion of melatonin. Given the vagus nerve consists of about 80% sensory afferent and 20% motor efferent fibers [31, 32], Meyers et al. demonstrated that selective afferent cervical VNS induces a marked and sustained increase in blood glucose level in anesthetized rats, while selective efferent cervical VNS did increase blood glucose levels temporarily without inhibiting insulin secretion [11]. Likewise, a recent study indicated that efferent vagal stimulation attenuates hyperglycemia in endotoxemia by inducing insulin in fasted mice [33]. However, while combined afferent and efferent cervical VNS impaired glucose tolerance and inhibited glucose-induced insulin secretion in conscious rats [12]. In contrast, cervical VNS of the intact vagus nerve at frequencies of 5 Hz and 14 Hz reduces blood glucose in diabetic rats by enhancing vagal efferent activity and the release of glucagon-like peptide-1, and the effect of intermittent stimulation is more powerful than continuous stimulation [14]. Current results demonstrated that cervical VNS increased FBG concentrations in non-diabetic subjects with commonly used stimulating parameters. These findings suggest that fibers within the cervical vagus nerve may differ in humans and animals, the specific vagus nerve fibers carry metabolic information both ascending to the CNS and descending to glycemic control organs may with different activation levels in animals and humans. While enticing, these data from animals and humans did not address the correspondence between stimulation of vagus nerve fiber subtypes and blood glucose regulation. To this end, specific electrodes and methodologies are needed for a new paradigm of stimulation of target-specific vagus nerve fibers to achieve the purpose of glycemic control.

It has been reported that vagus nerve blocking is often associated with weight loss in obese patients [23, 24]. Studies focusing on patients with epilepsy or depression who underwent VNS implantation demonstrated that patients experienced significant weight loss after long-term treatment [34, 35]. Moreover, the weight loss induced by VNS is positively associated with baseline BMI [35]. Similarly, data from animal models suggest that VNS actually induced weight loss but rather than preventing excess weight gain [36]. In the present study, the change in weight is inconsistent with the findings of the above studies. Since the exact mechanisms by which cervical VNS influences body metabolism and weight control remain incompletely understood, it is supposed that VNS may have a positive effect on appetite while relieving symptoms of epilepsy. In addition, the presence of seizures limits the activity of patients with epilepsy to a certain extent, thereby reducing energy metabolism and increasing in fat stores. Another source of possibility for the effect of VNS on weight is that stimulation of cervical vagus nerve disrupts vagal signaling both to and from the abdominal viscera related to gut motility and absorption. Future studies are warranted to elucidate the potential relationship between patient-specific VNS settings and weight control.

There are several limitations in the present study. First, as a retrospective study, it is impossible for us to do a very comprehensive analysis due to incomplete medical records. Due to the lack of hormonal data such as insulin, glucagon, and glucagon-like peptides-1, we cannot further explore the possible mechanism by which VNS can influence glycemic control. Second, the available data suggest that 12 months of cervical VNS raise FBG levels in epileptic patients. The magnitude of the glucose change is relatively small and may not be of clinical significance to patients with epilepsy; however this may not be true for diabetic patients. Although the FBG level is still within the normal range, we cannot answer the question if the FBG levels elevated following long-lasting chronic cervical VNS. Third, our study included patients receiving various AED, where the effect of AED on the glycemic control might be unpredictable. Finally, the findings in the current study are preliminary because they are based on a single-center, small sample size study without no-treatment control group. Since there was no difference in FBG levels between the sham-VNS and VNS groups at 4 months, the increase in FPG from baseline to 4 months is also likely due to factor other than VNS, and it is also likely the cause for the additional increase in FPG at 8- and 12-month follow-up periods. Future multicenter, sizeable, and prospective study should be conducted to explore the interaction between chronic cervical VNS and glycemic control.

## Conclusions

Our data demonstrate that electrical stimulation of vagus nerve at cervical level with commonly used parameters increases FBG concentrations in epileptic patients receiving VNS implantation. Moreover, the changes in FBG concentrations are negatively correlated with baseline FBG levels. While this is accompanied by the gains in weight, whether the elevation in FBG levels is causative physiology or sequela is unclear. Whether patients with long-term cervical VNS treatment are at greater risk of developing glucose intolerance and diabetes remains to be elucidated.

## Methods

### Study protocols

A randomized, double-blind controlled trial conducted in six centers to assess the efficacy and safety of 12 months of VNS in patients with drug-resistant epilepsy was retrospectively analyzed. The trial has been registered at clinicaltrials.gov protocol system (Clinical Trials Identifier: NCT02378792) and was updated in October 2016. Data from one center of the trial were selected for the present study to avoid the potential impact of VNS surgical procedures. At baseline period, subjects maintained a stable medication regimen for 4 weeks for seizure rate and basic clinical characteristics’ evaluation. Then, all participants were implanted with the VNS device (G111, PINS Medical, Ltd., Beijing, China) and randomized to either a VNS (*n* = 19) or sham control (*n* = 13) group. After the 2-week recovery period, the implanted pulse generators in the VNS group were turned on while those in the sham control group remained off. During the following 4-month double-blind period, the VNS group received patient-specific electrical stimulation and the sham control group received no stimulation. For the latter 8-month open-label period, subjects in the sham control group crossed over to receive personalized optimal stimulation. Both VNS and sham control groups were followed up to 12 months. Three months prior to the VNS surgery and during the 12-month follow-up period after the VNS treatment, the number and doses of the anti-epileptic drugs’ (AED) regimens, exercise, and eating habits were kept unchanged, and the patients or their family members were asked to keep seizure diaries to determine the baseline and 12-month follow-up seizure frequency. This study was approved by the Institutional Review Committee of Beijing Tiantan Hospital Capital Medical University, and all subjects, or parents/guardians of the subjects, gave informed consent in written form including for the collection of their information and usage for research. The methods in the study were carried out in accordance with the approved guidelines.

### Participants and enrollment

The patients had undergone VNS surgery at the Beijing Tian Tan Hospital, Capital Medical University between August 13, 2014 and December 31, 2014 and had finished their 12-month follow-up evaluation, and were included in the study. All patients underwent complete presurgical evaluations and neuropsychological assessments as part of their diagnoses to ascertain that their drug-resistant epilepsy was not suitable for traditional epileptic craniotomy surgery [37, 38]. Key inclusion criteria included individuals who (1) were 6- to 60-year old, (2) had tried at least two appropriate AED tested to tolerance or to blood levels at the upper end of the target range of which at least 2 had been tolerated at the normal dose, (3) had at least 7 seizures per month, (4) were in good health except for epilepsy, (5) with a minimum mental state examination (MMSE) score ≥ 18 (no severe cognitive impairment). Key exclusion criteria included (1) the results of medical imaging examination indicating that the epilepsy was caused by intracranial space-occupying lesions, (2) tumors, cardiopulmonary anomalies, diabetes, progressive neurological diseases, asthma, mental disease, or any other known diseases that may have affected the autonomic nervous system function, (3) alcohol addiction, smoking, and sleep-related breathing disorders, and (4) a history of medication that may have impacted the autonomic function and blood glucose levels.

### VNS treatment

The VNS system (G111, PINS Medical, Ltd., Beijing, China) was implanted under general anesthesia by an otolaryngologist. Spiral electrodes were placed around the left vagus nerve and connected to the programmable pulse generator subcutaneously. After VNS implantation, participants were randomized to either the VNS group (*n* = 19) or to the sham-VNS control group (*n* = 13). For the VNS group, pulse generator was turned on 2 weeks after implantation with initial settings being a current amplitude of 0.2 mA, frequency of 30 Hz, pulse width of 500 μs, stimulus on time of 30 s, and stimulus off time 5 min. Adjustments were made at intervals of about 2 weeks until the stimulation reached 1.0 mA. This was followed by 1-month intervals for the first 4 months and then preceded by 4-month intervals. At each follow-up visit, the output current was progressively increased by 0.2–0.3 mA until (1) the seizures were reduced by more than 50%, (2) the patient no longer tolerated the treatment, or (3) the current reached a maximum of 3.5 mA. In the sham-VNS control group, the output current was increased temporarily during each visit and switched off at the end of the visit. At the beginning of the open-label phase, the stimulation parameters of the sham-VNS control group were adjusted according to the procedure and parameters of the VNS group.

### Evaluations and outcome measurements

The primary outcome measure of the present study is the FBG concentrations in the morning measured at baseline, 4 months, 8 months, and 12 months. All FBG concentrations were measured by the glucose oxidase method. Secondary outcomes include body weight, BMI, and blood pressure assessed at baseline and all clinic visits within the 12-month follow-up period. In addition, demographic data, seizure type, epilepsy duration, etiology, age at VNS surgery, seizure frequency, number of AED used, total dose of AED per day, presurgical MRI or PET findings, ictal scalp video-EEG characteristic, and preoperative ECG recordings at baseline and the changes in severity of epilepsy at all clinical visits were observed.

### Statistical analysis

Data are presented as mean ± standard deviation (SD) for continuous variables. Gaussian distribution and homogeneity of variance tests were applied to determine the distribution and homoscedasticity of sample data. As a result of the non-normal distribution and heterogeneity of variance of some sample data, a Mann–Whitney *U* test was applied to compare the differences between VNS and sham-VNS groups. The Wilcoxon signed-rank test was used to compare the clinical variables of the 32 patients with drug-resistant epilepsy for the baseline and 4, 8, and 12 months after VNS treatment. For single predictive variable analysis using qualitative or categorical variables, Fisher’s exact tests were applied for comparison of the equality of proportions between the VNS and sham-VNS control groups. Multiple linear regression analyses were also employed to identify independent clinical factors related to fasting glucose levels and/or its changes at 4, 8, and 12 months’ follow-up. The goodness of fit of the test and reference regression line was quantified by the coefficient of determination *R*^2^. Partial correlation was applied to strengthen the relationship between FBG and stimulation amplitude while controlling for the effect of BMI. Statistical analyses were performed using SPSS version 20 software package (SPSS, Chicago, Ill, USA). All the *p* values were adjusted using the false discovery rate (FDR) method and a value of *p* < 0.05 was considered to indicate statistical significance.

## Supplementary information

**Additional file 1: Table S1.** Clinical data of 32 patients with drug-resistant epilepsy. AEDs, antiepileptic drugs; VPA, valproate; LTG, lamotrigine; CBZ, carbamazepine; OXCBZ, oxcarbazepine; LEV, levetiracetam; TPM, topiramate; PHB, phenobarbital; CZP, clonazepam; PHT, phenytoin; MGVPA, magnesium valproate; CPNCM, compound phenobarbital nitrazepam and chlorphenamine maleate; DZP, diazepam; ZNS, zonisamide; TCM: traditional Chinese medicine; PMT, primidone; GS, generalized seizure; FS, focal seizure. **Table S2.** Baseline clinical characteristics in the Sham-VNS and VNS groups. P values were provided for comparison between Sham-VNS and VNS groups at baseline. AED, antiepileptic drug; GS, generalized seizure; FS, focal seizure.

## Data Availability

The datasets used and/or analyzed during the current study are available from the corresponding authors F.M. (email: mengfangang@126.com) or W.W. (email: wang_weidong301@163.com) on reasonable request.

## Abbreviations

VNS: Vagus nerve stimulation
FBG: Fasting blood glucose
BMI: Body mass index
AED: Anti-epileptic drugs
MMSE: Minimum mental state examination
SD: Standard deviation
FDR: False discovery rate
ANS: Autonomic nervous system
CNS: Central nervous system
taVNS: Transcutaneous auricular vagus nerve stimulation
HbA1C: Glycosylated hemoglobin
