# Supplementary material for: Chronic vagus nerve stimulation for drug-resistant epilepsy may influence fasting blood glucose concentration
Source: Biomed Eng Online. 2020 May 29;19:40. doi: 10.1186/s12938-020-00784-1 (PMC7257242; doi:10.1186/s12938-020-00784-1)
Supplement: Supplementary file 1 — Additional file 1: Table S1. Clinical data of 32 patients with drug-resistant epilepsy. AEDs, antiepileptic drugs; VPA, valproate; LTG, lamotrigine; CBZ, carbamazepine; OXCBZ, oxcarbazepine; LEV, levetiracetam; TPM, topiramate; PHB, phenobarbital; CZP, clonazepam; PHT, phenytoin; MGVPA, magnesium valproate; CPNCM, compound phenobarbital nitrazepam and chlorphenamine maleate; DZP, diazepam; ZNS, zonisamide; TCM: traditional Chinese medicine; PMT, primidone; GS, generalized seizure; FS, focal seizure. Table S2. Baseline clinical characteristics in the Sham-VNS and VNS groups. P values were provided for comparison between Sham-VNS and VNS groups at baseline. AED, antiepileptic drug; GS, generalized seizure; FS, focal seizure. [file 12938_2020_784_MOESM1_ESM.docx]

| **Patient ID** | **Gender** | **Age (year)** | **Number of AEDs** | **AEDs and daily dose (mg)** | **Ictal scalp EEG characteristics** | **Seizure types** | **Etiology** |
| --- | --- | --- | --- | --- | --- | --- | --- |
| **P1** | M | 19 | 3 | CBZ(900), VPA(1000), PHT(450) | Bilateral temporal abnormalities | GS, FS | Cryptogenic |
| **P2** | M | 11 | 3 | VPA(1000), CBZ(600), TPM(50) | Left parietal, occipital, temporal focus | FS | Symptomatic (left occipital lobe atrophy) |
| **P3** | M | 7 | 4 | TPM(12.5), VPA(500), CZP(1.25), LTG(100) | Bilateral frontal, temporal focus | GS, FS | Cryptogenic |
| **P4** | M | 19 | 1 | LEV(1000) | Left temporal focus | FS | Cryptogenic |
| **P5** | F | 27 | 4 | LEV(1500), PHB(90), LTG(200), VPA(1000) | Bilateral frontal, temporal focus | GS, FS | Symptomatic (postencephalitis) |
| **P6** | M | 22 | 2 | OXCBZ(900), LEV(1000) | Bilateral frontal, temporal focus | GS, FS | Symptomatic(white matter myelination) |
| **P7** | M | 33 | 1 | CBZ(600) | Bilateral temporal abnormalities | GS, FS | Symptomatic (right hippocampal atrophy) |
| **P8** | M | 21 | 7 | MGVPA(500), DZP(20), PHT(300), OXCBZ(900), LTG(150), TCM(2800) | Bilateral frontal, temporal focus | GS | Cryptogenic |
| **P9** | F | 24 | 3 | LTG(200), LEV(2000), OXCBZ(1500) | Bilateral temporal abnormalities | GS | Symptomatic (encephalomalicia) |
| **P10** | F | 28 | 2 | VPA(1000), LTG(150) | Bilateral temporal, occipital focus | GS | Cryptogenic |
| **P11** | F | 19 | 1 | VPA(1000) | Left frontal, parietal; Right temporal, parietal focus | GS | Symptomatic (encephalomalicia & craniocerebral trauma) |
| **P12** | M | 27 | 2 | PHB(180), CBZ(1800) | Non-specific EEG abnormalities | GS | Cryptogenic |
| **P13** | F | 25 | 1 | VPA(400) | Left occipital, bilateral temporal abnormalities | GS, FS | Symptomatic (right hippocampal atrophy) |
| **P14** | M | 18 | 2 | LEV(250), VPA(1000) | Right temporal abnormalities | GS, FS | Symptomatic (left hippocampal sclerosis) |
| **P15** | F | 19 | 4 | CBZ(1100), LEV(2000), PHB(45), LTG(150) | Bilateral frontal, parietal, temporal abnormalities | GS, FS | Symptomatic (postencephalitis) |
| **P16** | M | 7 | 1 | CBZ(400) | Left parietal focus | FS | Symptomatic (tuberous sclerosis) |
| **P17** | M | 32 | 1 | VPA(1500) | Non-specific EEG abnormalities | GS | Cryptogenic |
| **P18** | M | 21 | 1 | VPA(1500) | Right temporal focus | GS, FS | Symptomatic (encephalomalicia) |
| **P19** | M | 38 | 2 | CBZ(600), MGVPA(500) | Right temporal, bilateral frontal abnormalities | GS, FS | Symptomatic(postoperative glioma) |
| **P20** | F | 21 | 1 | CBZ(800) | Right parietal, temporal focus | FS | Cryptogenic |
| **P21** | M | 31 | 2 | MGVPA(1000), CPNCM(1000) | Right frontal, temporal abnormalities | GS, FS | Symptomatic (encephalomalicia) |
| **P22** | M | 12 | 2 | OXCBZ(450), ZNS(300) | Non-specific EEG abnormalities | GS, FS | Symptomatic (heterotopias) |
| **P23** | M | 25 | 2 | CBZ(200), LTG(50) | Right frontal, parietal, temporal abnormalities | GS, FS | Symptomatic(pachygyria abnormality) |
| **P24** | M | 10 | 3 | LTG(62.5), LEV(1500), VPA(18) | Non-specific EEG abnormalities | FS | Cryptogenic |
| **P25** | F | 7 | 3 | LEV(750), VPA(500), TPM(150) | Bilateral temporal, Right parietal focus | GS | Symptomatic (encephalomalicia) |
| **P26** | F | 11 | 2 | LTG(250), OXCBZ(450) | Non-specific EEG abnormalities | GS, FS | Cryptogenic |
| **P27** | F | 10 | 4 | LTG(50), CPNCM(600), PMT(250), MGVPA(500) | Left temporal abnormalities | FS | Symptomatic (left hippocampal abnormalties) |
| **P28** | M | 15 | 2 | CBZ(800), VPA(2000) | Left parietal, occipital, Bilateral temporal focus | GS, FS | Symptomatic (toxicosis) |
| **P29** | M | 6 | 3 | VPA(750), LTG(100), TPM(150) | Left frontal, parietal, temporal focus | FS | Symptomatic (focal cortical dysplasia) |
| **P30** | M | 34 | 1 | CBZ(1400) | Bilateral temporal, frontal abnormalities | GS, FS | Symptomatic (encephalomalicia & atrophy) |
| **P31** | F | 10 | 3 | OXCBZ(375), ZNS(200), CZP(2) | Non-specific EEG abnormalities | FS | Cryptogenic |
| **P32** | M | 7 | 4 | OXCBZ(900), TPM(150), PHB(60), CZP(3) | Non-specific EEG abnormalities | GS, FS | Cryptogenic |

Table S1. Clinical data of 32 patients with drug-resistant epilepsy. AEDs, antiepileptic drugs; VPA, valproate; LTG, lamotrigine; CBZ, carbamazepine; OXCBZ, oxcarbazepine; LEV, levetiracetam; TPM, topiramate; PHB, phenobarbital; CZP, clonazepam; PHT, phenytoin; MGVPA, magnesium valproate; CPNCM, compound phenobarbital nitrazepam and chlorphenamine maleate; DZP, diazepam; ZNS, zonisamide; TCM: traditional Chinese medicine; PMT, primidone; GS, generalized seizure; FS, focal seizure.

| **Variables** | **Sham-VNS, n=13** |  | **VNS, n=19** | **Pooled, n=32** | **p** |
| --- | --- | --- | --- | --- | --- |
| **Demographic data** | | | | | |
| Male/Female | 9/4 |  | 12/7 | 21/11 | 1.000 |
| Age (years) | 19±8 |  | 20±10 | 19±9 | 0.817 |
| **AEDs information** |  |  |  |  |  |
| Number of AEDs | 3±2 |  | 2±1 | 2±1 | 0.843 |
| Daily dose (mg) | 1547±1097 |  | 1453±845 | 1491±939 | 0.908 |
| **Seizure characteristics** | | | | | |
| Epilepsy duration (years) | 10±6 |  | 12±10 | 11±8 | 0.802 |
| Seizures per month | 79±137 |  | 124±233 | 106±198 | 0.155 |
| FS | 3 (23.1%) |  | 5 (26.3%) | 8 (25.0%) | 1.000 |
| GS | 2 (15.4%) |  | 5 (26.3%) | 7 (21.9%) | 0.671 |
| GS+FS | 8 (61.5%) |  | 9 (47.4%) | 17 (53.1%) | 0.491 |
| **Etiology** |  |  |  |  |  |
| Symptomatic | 7 (53.8%) |  | 13 (68.4%) | 20 (62.5%) | 0.473 |
| Cryptogenic | 6 (46.2%) |  | 6 (31.6%) | 12 (37.5%) | 0.473 |

Table S2. Baseline clinical characteristics in the Sham-VNS and VNS groups. P values were provided for comparison between Sham-VNS and VNS groups at baseline. AED, antiepileptic drug; GS, generalized seizure; FS, focal seizure.
